# Possible mechanism of schizophrenia origin by excess GABA and synaptic pruning

**DOI:** 10.1016/j.ibneur.2023.07.005

**Published:** 2023-07-28

**Authors:** A. Rabinovitch, D. Braunstein, R. Rabinovitch, Y. Biton

**Affiliations:** a Physics Dept. Ben-Gurion University, Beer-Sheva, Israel; b Physics Dept. Sami Shamoon College of Engineering, Beer-Sheva, Israel; c Makif YudAlef, Rishon Lezion, Israel

**Keywords:** Schizophrenia, GABA synaptic pruning

## Abstract

Schizophrenia is a psychotic disorder that affects approximately 1% of the global population. However, the etiology of this illness remains a subject of debate. One of the proposed mechanisms underlying schizophrenia is the synaptic pruning mediated by microglia in the brains of individuals with schizophrenia, although the precise mechanisms of this process remain elusive. In this regard, we propose that the potential development of the disease stems from both a genetic predisposition leading to an excessive production of GABAergic neurons and an exaggerated effort to maintain the E/I (excitation/inhibition) balance in the brain.

## Introduction

Schizophrenia is a chronic psychiatric disorder that is associated with significant morbidity and early mortality. It is characterized by symptoms such as hallucinations, delusions, disturbed thinking, and behavioral problems (Bartoli et al., 2019). The global incidence of schizophrenia is approximately 1%. Since the 1990 s, the concept of synaptic pruning as a potential cause of schizophrenia has garnered considerable attention (Feinberg, 1990, Keshavan et al., 1994, Miyanishi et al., 2021). However, understanding the underlying mechanism of this process and why it affects certain individuals and not others has remained elusive. In this study, we propose that a possible fundamental factor contributing to the development of schizophrenia is of genetic origin, specifically related to the production of an excessive number of GABAergic neurons encoded in the genetic makeup of individuals at risk of developing the disorder.

In a normal brain, there exists a delicate balance between the populations of GABAergic and glutamatergic neurons in the central nervous system, known as the excitatory-inhibitory (E/I) balance. The typical balanced E/I ratio is approximately 4, with around 80% glutamatergic neurons and 20% GABAergic neurons (see, for example, (Sukenika et al., 2021)). We hypothesize that a genetic deviation from this ratio could potentially lead to schizophrenia by affecting the activity of microglia.

In addition to the vast number of neurons, the brain also contains white matter and glial cells. Microglial cells, a type of glia, play a role in patrolling the brain tissue and regulating synaptic numbers by either pruning synapses or promoting their formation (Schafer et al., 2012, Miyamoto et al., 2016).

To investigate the genetic E/I ratio, researchers have utilized induced pluripotent stem cells (iPSCs) derived from individuals diagnosed with schizophrenia, while measurements of the adult ratio have been obtained from postmortem examinations of similar patients. Induced pluripotent stem cells were first generated from human fibroblasts in 2006, using factors such as OCT4, SOX2, KLF4, and c-MYC (Takahashi and Yamanaka, 2006). These iPSCs can differentiate into various cell types, including neurons and glial cells, and they retain the exact genetic code of the individual from whom they were derived. Consequently, iPSCs have been instrumental in modeling complex neuropsychiatric disorders with a genetic and phenotypic basis.

In a recent study utilizing iPSCs (Sawada et al., 2020), the measurement of genes associated with GABAergic neurons in pluripotent stem cells derived from individuals with schizophrenia revealed that the numbers of these genes were *much higher* than the quantity needed for a normal E/I balance. Conversely, postmortem measurements of neuron numbers in schizophrenic patients indicate a deficiency of both GABAergic and glutamatergic neurons (Birur et al., 2017), which could potentially be the root cause of the disease. Recent studies have specifically identified these deficits in layer 3 of the dorsolateral prefrontal cortex (Hoftman et al., 2018), while other layers of this brain region exhibit elevated levels of glutamatergic neurons and unchanged levels of GABAergic neurons compared to non-schizophrenic subjects (Dienel et al., 2020).

How can we explain this discrepancy, particularly in layer 3? Could it be attributed to an initial surplus of GABAergic genes that eventually leads to a postmortem decrease in both GABAergic and glutamatergic cells? We propose that the initial excess of GABAergic neurons in the brain, driven by genetic factors, may act as a triggering factor that ultimately results in reduced neural expression of both neuron types, possibly due to an overactive function of microglia.

## The model

Most researchers use the terms "E/I ratio" and the ratio between the number of glutamatergic neurons and GABAergic neurons interchangeably. However, it is important to note that the E/I ratio measures the relative "power" or excitability of the group of glutamatergic neurons compared to the group of GABAergic neurons, taking into account not only the number of neurons but also their ability to influence other neurons through synaptic connections. Therefore, alterations in the E/I ratio can occur as a result of synaptic pruning carried out by microglia, similar to the effects of neuronal pruning. It is worth mentioning that other factors, unrelated to synaptic pruning, can also influence the power of neuronal groups and the E/I ratio. These factors include the number and function of GABA receptors, glutamatergic receptors, the number of postsynaptic spines, presynaptic boutons, and the geometries of neurons. However, these factors are beyond the scope of this study.

We have developed a mathematical model to describe a scenario in the schizophrenic brain where the original genetic balance between excitatory and inhibitory neurons (referred to as the E/I balance) is heavily skewed towards the excess of GABAergic neurons, while in the adult schizophrenic brain, two possibilities are observed:

1) The E/I ratio is balanced, but both the power of GABAergic neurons and glutamatergic neurons are significantly diminished.
2) The E/I balance is tipped towards the glutamatergic side.

Before delving into the explicit calculations, it is important to note that in a normal E/I balance (as described in various studies), the power of excitatory (glutamatergic) neurons constitutes approximately 80% of the total power in the central nervous system, while the power of inhibitory (GABAergic) neurons accounts for approximately 20% of the total power. In the following discussion, we will refer to these fractions or percentages as "E" and "I," respectively.

Our model postulates that in patients with schizophrenia, the initial power of GABAergic neurons after birth is increased beyond the necessary power to maintain the equilibrium E/I balance by a fraction C represented as $\left(1+C\right)\times I$.

**Fig. 1:**
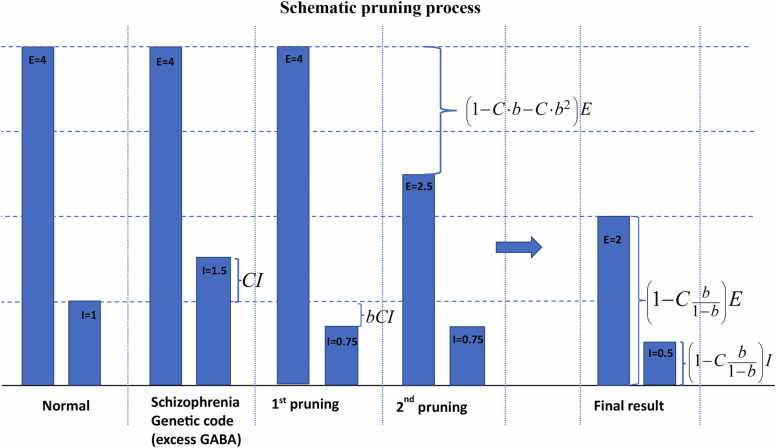
The pruning process (schematic). E and I powers are depicted during its development. Normal powers are shown in the first frame while increased GABAergic power (by a factor C, here taken as 50%) controlled by the genetic code in schizophrenia patients is shown in the second frame. The two initial pruning stages (overexpressed by a factor b here taken as 0.5) are shown in the next two frames. This process continues until the final power balance depicted in the last frame (the possible situation in schizophrenia patients) is approximately reached. Note that the values of C and b used here are only for demonstration purposes. Actual values are possibly smaller. Note also that the E/I ratio in both the normal and the final stages is 4:1.

Let us consider the response of glial cells (GCs), which are responsible for maintaining homeostatic equilibrium, to this imbalance. These cells, referred to as "C units" of the brain's complement system (Dalakas et al., 2020, Andoh et al., 2019), and/or microglia, attempt to rectify the situation by eliminating the excess cells or their synapses (Dalakas et al., 2020, Clarkson, 2010). However, due to their overactivity, instead of eliminating the precise fraction of cells required (C), they eliminate an exaggerated fraction (aC), where a is greater than 1. As a result, the E/I balance tilts towards the glutamatergic neurons. Subsequently, the GCs excessively reduce the power of glutamatergic neurons. This process continues until a new balance is eventually reached, but with diminished powers in both groups of neurons. The developmental stages of this process, illustrated schematically in Fig. 1, are as follows.

1. The power of normal neurons is represented by the fractions E (∼80%) and I (∼20%).
2. The initial ratio of fractions for a patient with schizophrenia, determined by their genetic code, is given by: $E:\left(1+C\right)\times I$, which means that the neuronal E/I ratio is: $80\%:\left(1+C\right)\times 20\%$.
3. The fractions ratio after the first GCs operation, which cuts a portion aC of the GABAergic power, is: $E:\left(1+C-\mathrm{aC}\right)\mathrm{xI}$ or $E:\left(1-\mathrm{bC}\right)\mathrm{xI}\;\left(\mathrm{where}\;b=a-1\right),i.e.,\mathrm{the}\;E/I\;ratio is\;\mathrm{is}\;:80\%:\left(1-\mathrm{bC}\right)\;x20\%$.
4. If equilibrium is now sought for by the GCs operation by decreasing the Glutamatergic neurons power fraction and the GCs operation is again in excess by the same magnitude, then the next fractions ratio is: $\left(1-\mathrm{abC}\right)\mathrm{xE}:\left(1-\mathrm{bc}\right)\mathrm{xI},\mathrm{or}\;\left(\mathrm{recall}\;\mathrm{that}\;a=1+b\right),\left(1-\mathrm{bC}-b^{2}C\right)\mathrm{xE}:\left(1-\mathrm{bc}\right)\mathrm{xI}$.
  Thus, the positive GABAergic fraction power excess after this stage is $b^{2}C$
5. In the next stage the GCs reduces this fraction by $ab^{2}C$, so the next fractions ratio is:
  $$\left(1-\mathrm{bC}-b^{2}C\right)\mathrm{xE}:\left(1-\mathrm{bC}-b^{2}C-b^{3}C\right)\mathrm{xI}$$
6. Each bracketed side of this E/I ratio is 1 minus a geometric series. The first term of the geometric series is bC and its constant ratio is *b*. If the process continues, and if *b* is smaller than 1, the final fractions ratio will be 1 minus the sum of the series which is $C\frac{b}{1-b}$, namely: $1-C\frac{b}{1-b}:1-C\frac{b}{1-b}\;$or a fraction of $1-C\frac{b}{1-b}$ for both neuron types.

$$\mathrm{The E}/\mathrm{I ratio therefore becomes}:\left(1-C\frac{b}{1-b}\right)x\;E:\left(1-C\frac{b}{1-b}\right)x\;I$$

Note that we have used the sum of an *infinite* series when life is limited. This use is allowed here since we assume that *b* is < < 1 and therefore the series convergence is fast. The final stage of the brain may not reach this ultimate power but approaches it closely.

Results show that both the GABAergic and the Glutamatergic neurons powers have decreased approximately *D* times their normal quantity, where $D=C\frac{b}{1-b}$, the *fraction-decrease.*

**Fig. 2:**
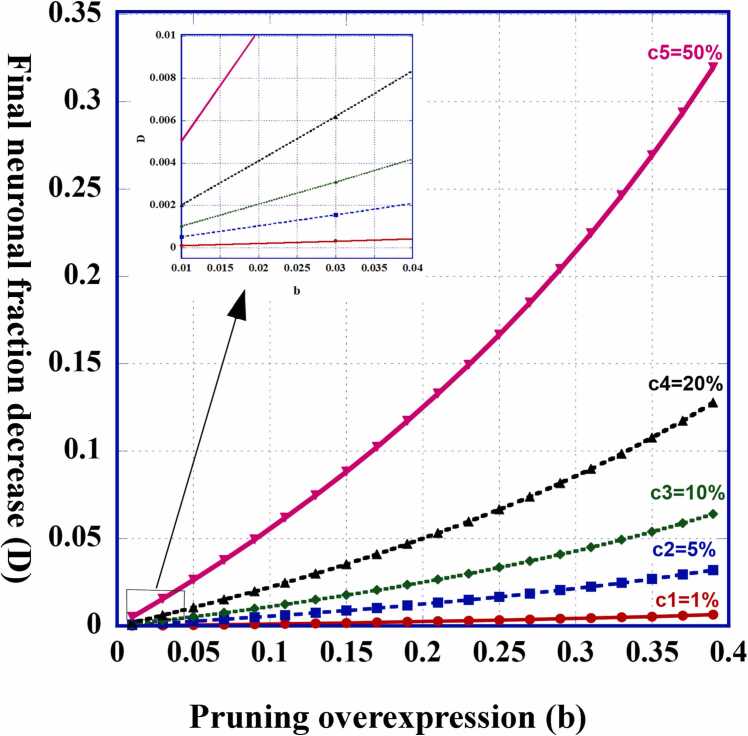
Compared to Fig. 1 final result, both E and I powers are reduced by a fraction $D=C\frac{b}{1-b}$. It depicts this final power reduction fraction of neurons of both modes as a function of the fraction of microglial "over-functioning", for several values of *C* – the initial genetically encoded excess percentage of GABAergic neurons. Inset: enlarged picture of the cases of small b values.

Fig. 2 depicts the final power fraction decrease (*D*) as a function of the "excess" or overexpression of the GC function (*b*), for different values of *C*, the initial genetic GABAergic excess power percentage. It is seen, that even if *b* is of the order of 10%, for *D* to be "significant" (say, higher than 0.5%), *C* should be higher than 4.5%. Since exact results for *D* or *C* in the current existing literature are very scattered (Zhang et al., 2020) or controversial, a real estimate of *b* is presently unachievable.

## Discussion

Different measurements (e.g., (Grent-'t-Jong et al., 2018)) indicate that, a change of the E/I balance in the brain towards increased excitation, is the cause of schizophrenia at onset. This situation can be due to the model discussed above after only the first GCs’ operation, namely when the E/I ratio is E:(1−bC)xI, (see Fig. 1) or when the GABAergic neuron power is reduced by bC relative to their normal ratio. This finding corresponds to the observations in Lui et al. (2016), which suggests a malfunction in the pruning process. Furthermore, according to Grent-'t-Jong et al. (2018), in the later stages of schizophrenia: "chronic schizophrenia patients showed widespread decreased low gamma-band activity in frontal, temporal and sensorimotor areas", indicating lower values of both GABAergic and Glutamatergic powers, as predicted by the situation at the *end* of the GC process discussed here.

Most of the scientific papers that investigate microglia pruning focus on abnormalities in the E/I balance, as observed in Lui, 2016, Koyama and Ikegaya, 2015a, which can be caused by malfunctioning pruning processes. However, it is evident that the E/I balance is typically preserved in the majority of individuals, including both humans and animals. This observation suggests the existence of a mechanism, as described in Salter and Stevens (2017), that ensures the preservation of this balance during the pruning process.

Regarding the involvement of microglia in maintaining homeostasis, even in individuals without pathological conditions, reference (Koyama and Ikegaya, 2015b) and the cited references therein provide valuable insights. The authors of this reference highlight that recent studies have revealed the significant role of microglia not only in inflammatory processes but also in the normal functioning of the brain. Specifically, microglia have been shown to preferentially engulf and eliminate weaker or less active synapses, thereby contributing to the development of well-defined neural circuits that consist of stronger and more active synapses. This emphasizes the essential contribution of microglia in shaping and refining functional neural circuits, even in the absence of disease.

In the present case, although, according to the genetic code, the GABAergic neuron power is too high, their synapses could be pruned in such a way that their *power* becomes ∼20% of the total power of synapses. We also assume, that neurons, whose synapses are disproportionately reduced, can become apoptotic and engulphed by the microglia (Witting et al., 2000; Morini et al., 2021). Such a process can explain neuronal *number* decrease following synapse pruning. The "excessive" process, described in detail in the model section, can be attributed to both the synapse-pruning process and the neuron-reduction process.

It is not clear when, during the patient's life, these processes of developing an excess of GABAergic power and the subsequent pruning and phagocytosis take place. It is conceivable that the first synapse-pruning stage takes place even pre-birth, while the neuron-reduction (or synapse-reduction) stage occurs during the adulthood period (Salter and Stevens, 2017). Such a sequence could explain the normal behavior of schizophrenia patients during childhood and the outbreak of the disorder's symptoms during adulthood.

Since schizophrenic patients are not recognized at birth, no E/I measurements can be carried out on the same patient throughout life. If we could measure the E/I ratio and the total neuron powers of many people throughout life, we would eventually have such results also for schizophrenic patients. But, since the abundance of such patients in the general population is only ∼1%, and since these patients are unknown before the disorder’s outbreak, it seems a difficult task, even if this percentage is increased by choosing the cohorts for the experiment by a known familial schizophrenia incidence.

For a long time, brain inflammation has been considered as a cause for schizophrenia (Morini et al., 2021), where this process in the brain is partially carried out by the microglia. Furthermore, positive correlations were observed between microglial activations and schizophrenia symptoms and durations (Müller et al., 2015). Thus, the activation of microglia in the above-mentioned E/I balancing mechanism can possibly be understood as being similar to an inflammation procedure.

Can such *excess pruning* as is discussed here occur for non-schizophrenic persons? And if so, why is this process not a harmful one? In other words, does the excess activity of the microglial cells, which leads to a decrease of the total neuronal power by a fraction of $\frac{b}{1-b}$, operate also during "regular small" malfunctions that tip the E/I balance in either direction? And, if so, why the total neural power does not decrease? The answer depends on the values of *C* and *b*. If we assume (Fig. 2) that *b* is of the order of 5% and the regular *C* values which can occur in normal brains, are less than 1%, then, if even the return to homeostasis is carried out in excess, the total neural power would decrease, but by only $5x\;10^{-4}\;$, which is hardly observable and probably does not lead to any harmful consequences.

## Conclusion

A simple mathematical model of proposed excess pruning mechanism was shown to explain a conceivable schizophrenic origin, by following the possible E/I ratio from its tilted towards GABAergic value at the person's conception to the final value at adulthood. If, under additional experimental research, our approach is fully confirmed, possible interventions in the natural pruning process would be called for to treat this malfunction.

## Funding

Not Applicable.

## CRediT authorship contribution statement

Professor Avinoam Ravitch conceived the study. Mrs. Revital Rabinovitch did the programing. All the authors contributed equally in the calculations and writing.

## Declaration of Competing Interest

We the authors of the current manuscript declare no competing interest.

## References

[bib1] Andoh M., Ikegaya Y., Koyama R. (2019). Synaptic pruning by microglia in epilepsy. J. Clin. Med..

[bib2] Bartoli F. (2019). Adjunctive second-generation antipsychotics for specific symptom domains of schizophrenia resistant to clozapine: a meta-analysis. J. Psychiatr. Res..

[bib3] Birur B. (2017). Brain structure, function, and neurochemistry in schizophrenia and bipolar disorder-a systematic review of the magnetic resonance neuroimaging literature. NPJ Schizophr..

[bib4] Clarkson A.N. (2010). Reducing excessive GABA-mediated tonic inhibition promotes functional recovery after stroke. Nature.

[bib5] Dalakas M.C., Alexopoulos H., Spaeth P.J. (2020). Complement in neurological disorders and emerging complement-targeted therapeutics. Nat. Rev. Neurol..

[bib6] Dienel S.J. (2020). Markers of glutamate and GABA neurotransmission in the prefrontal cortex of schizophrenia subjects: disease effects differ across anatomical levels of resolution. Schizophr. Res..

[bib7] Feinberg I. (1990). Cortical pruning and the development of schizophrenia. Schizophr. Bull..

[bib8] Grent-'t-Jong T. (2018). Resting-state gamma-band power alterations in schizophrenia reveal E/I-balance abnormalities across illness-stages. Elife.

[bib9] Hoftman G.D. (2018). Altered gradients of glutamate and gamma-aminobutyric acid transcripts in the cortical visuospatial working memory network in schizophrenia. Biol. Psychiatry.

[bib10] Keshavan M.S., Anderson S., Pettegrew J.W. (1994). Is schizophrenia due to excessive synaptic pruning in the prefrontal cortex? The Feinberg hypothesis revisited. J. Psychiatr. Res..

[bib11] Koyama R., Ikegaya Y. (2015). Microglia in the pathogenesis of autism spectrum disorders. Neurosci. Res..

[bib12] Koyama R., Ikegaya Y. (2015). Microglia in the pathogenesis of autism spectrum disorders. Neurosci. Res..

[bib13] Lui H. (2016). Progranulin deficiency promotes circuit-specific synaptic pruning by microglia via complement activation. Cell.

[bib14] Miyamoto A. (2016). Microglia contact induces synapse formation in developing somatosensory cortex. Nat. Commun..

[bib15] Miyanishi K. (2021). Synaptic elimination by microglia and disturbed higher brain functions. Neurochem. Int..

[bib16] Morini R. (2021). Strategies and tools for studying microglial-mediated synapse elimination and refinement. Front Immunol..

[bib17] Müller N. (2015). The role of inflammation in schizophrenia. Front. Neurosci..

[bib18] Salter M.W., Stevens B. (2017). Microglia emerge as central players in brain disease. Nat. Med..

[bib19] Sawada T. (2020). Developmental excitation-inhibition imbalance underlying psychoses revealed by single-cell analyses of discordant twins-derived cerebral organoids. Mol. Psychiatry.

[bib20] Schafer D.P. (2012). Microglia sculpt postnatal neural circuits in an activity and complement-dependent manner. Neuron.

[bib21] Sukenika N., Vinogradovb O., Weinreba E. (2021). Neuronal circuits overcome imbalance in excitation and inhibition by adjusting connection numbers. PNAS.

[bib22] Takahashi K., Yamanaka S. (2006). Induction of pluripotent stem cells from mouse embryonic and adult fibroblast cultures by defined factors. Cell.

[bib23] Witting A. (2000). Phagocytic clearance of apoptotic neurons by Microglia/Brain macrophages in vitro: involvement of lectin-, integrin-, and phosphatidylserine-mediated recognition. J. Neurochem..

[bib24] Zhang W. (2020). Brain gray matter network organization in psychotic disorders. Neuropsychopharmacology.

